# Mapping Theoretical Frameworks of Positive Psychology in Second Language Acquisition Research: A Scoping Review

**DOI:** 10.1002/pchj.70110

**Published:** 2026-06-07

**Authors:** Kenan Gao, Juan Zhang

**Affiliations:** ^1^ Faculty of Education University of Macau Macau China; ^2^ Centre for Cognitive and Brain Sciences University of Macau Macau China

**Keywords:** positive psychology, PRISMA‐ScR, scoping review, second language acquisition, theoretical framework

## Abstract

Positive psychology (PP) has become a key perspective in second language acquisition (SLA), with growing attention to learners' emotions, motivation, engagement, well‐being, and self‐regulation. However, existing PP–SLA studies have not been sufficiently mapped in terms of how theoretical frameworks are selected, classified, and operationalized. This scoping review aims to map and classify publication characteristics (e.g., geographic distribution, journal rankings) and the theoretical frameworks and models that have guided the application of PP in SLA. Following PRISMA extension for Scoping Reviews (PRISMA‐ScR), the review was conducted in two stages. First, review, theoretical, and conceptual articles published between 2021 and 2025 were screened to identify higher‐level theoretical discussions in PP–SLA research. Second, empirical studies published in 2025 were examined to capture the most recent operational applications of these frameworks. A total of 38 studies met the inclusion criteria, comprising 6 review articles and 32 empirical studies. Findings indicate that PP functions as the broad conceptual umbrella of the field, while Broaden‐and‐Build Theory, Control‐Value Theory, Grit Theory, Flow Theory, Self‐Determination Theory, and Social Cognitive Theory represent the most frequently used specific theories/models. The review further classifies the seven major frameworks into four higher‐order categories: well‐being and flourishing frameworks, emotion‐centered theories, persistence and engagement theories, and motivational regulation theories. This scoping review contributes to PP–SLA research by moving beyond a list of individual theories and offering a hierarchical classification of theoretical framework use in the field. It also identifies key gaps concerning age‐sensitive theorization, digital and non‐formal learning contexts, cross‐cultural applicability, teacher‐related dimensions, and the need for stronger longitudinal and experimental designs.

## 1 Introduction

*What a man can be, he must be. This need we call self‐actualization*. Maslow (1943)

Positive Psychology (PP) remains committed to enabling the flourishing of individuals, communities, and society (Seligman and Csikszentmihalyi 2000). It is a paradigm shift from a historical emphasis on human shortcomings to an emphasis on understanding, developing, and promoting human strengths (Seligman and Csikszentmihalyi 2000). PP is deeply rooted in the traditions of humanistic psychology (MacIntyre and Mercer 2014), and its intellectual foundations can be traced back to ancient Eastern and Western philosophical traditions that have long grappled with the question of what it means to live a good and meaningful life (e.g., love, virtue, and character strength) (Dahlsgaard et al. 2005). In the more contemporary history of psychology and education, humanistic tradition has made enduring contributions to the development of PP (Maslow 1979). According to Snyder et al. (2009), the topics and concepts that have been identified in PP are presented in Table 1.

**TABLE 1:** Topics and concepts in PP (Snyder et al. 2009).

positive emotions	character strengths	future‐mindedness
emotional intelligent	compassion	capacity for pleasure
flow	humility	honesty
curiosity and interest	meaning in life	personality
resilience	self‐determination	wisdom
self‐esteem	subjective well‐being	mindfulness
happiness	optimism	creativity
love	toughness	realism
hope	rationality	insight
forgiveness	interpersonal skill	positive ethics
self‐efficacy	perseverance	reality negotiation
courage	gratitude	personal control
finding goals in life	empathy	uniqueness seeking

The humanistic movement peaked in language education during the 1970s and 1980s (MacIntyre and Mercer 2014). Its essence was an integrated view of learners, encompassing both cognitive and affective dimensions of learning (MacIntyre and Mercer 2014). With the humanistic development of PP and language education, MacIntyre and Gregersen (2012) brought PP to applied linguistics and realized a paradigm shift. In recent years, PP has been widely recognized in second language acquisition (SLA), serving as a “positive turn” in SLA research (Dewaele et al. 2019; Padilla et al. 2020). It is an indication of growing interest in enhancing both learners' well‐being and second or foreign language (henceforth L2) achievement (Oxford 2016). Traditionally, there have been relatively few representations of positive emotions in SLA studies, as these have previously focused on anxiety (Gao et al. 2026), among other negative affective traits. Diverging considerably from past frameworks, this shift revived the recognition of language learners' affective state (Li et al. 2018).

Both theories and empirical evidence have emphasized the role of PP in SLA. Theoretically, this movement for SLA is underpinned by major theories, such as Fredrickson's (2001) broaden‐and‐build theory and Pekrun's (2006) control‐value theory, which both identify crucial roles for positive emotions within the learning process (MacIntyre and Gregersen 2012). These theories have emphasized an expanding empirical literature examining L2 learners' affective experiences. Empirically, Lake (2013) was a pioneering figure in explicitly integrating concepts drawn from PP into SLA, with emphasis on Japanese learners' positive self‐concept, positive L2 self, self‐efficacy, and intended learning effort. Lake's (2013) research presented empirical evidence that constructs based on PP are strongly linked to learners' motivational constructs and to L2 performance (measured by TOEIC Bridge in that study). More recently, Wang and Liu (2023) carried out a meta‐analysis to synthesize the relationship between emotional intelligence and L2 learning. Based on 39 independent research studies with a total of 6571 participants, the meta‐analysis identified a moderate‐to‐large, positive correlation (*r* = 0.43, 95% CI: 0.32, 0.53) between learners' EI and actual L2 achievement.

However, existing PP–SLA research remains insufficiently organized in terms of the selection, classification, and operationalization of theoretical frameworks. To establish a clearer theoretical map of the intersection between PP and SLA, this scoping review aims to map, classify, and synthesize PP‐related theoretical frameworks and/or models employed in SLA research.

Specifically, this scoping review addresses the following questions:

1. What theoretical frameworks and models have been used in recent PP–SLA research?
2. How can these frameworks be classified according to their conceptual scope and explanatory mechanisms?
3. How have major theoretical frameworks been operationalized in SLA contexts?

## 2 Method

This scoping review was conducted and reported with reference to the PRISMA extension for Scoping Reviews (PRISMA‐ScR; Tricco et al. 2018). Scoping reviews are well‐suited to broad and conceptually diverse research areas because they offer a structured yet flexible approach to mapping the extent, range, and nature of existing literature and identifying gaps for future inquiry (Arksey and O'Malley 2005). This approach is appropriate for the present study because it provides a suitable methodological basis for synthesizing patterns of theoretical use, developing a higher‐order classification of major frameworks, and identifying directions for future research.

### 2.1 Literature Search

The search process had four phases: (1) Identification stage: keywords were derived from previously published literature in PP and related words. (2) Screening stage: searched literature was screened based on predefined inclusion and exclusion criteria. (3) Eligibility stage: assessment of chosen papers against study purpose. (4) Inclusion stage: making final judgments about which papers to include within the review.

The literature search was performed on April 9th, 2025, in the following four databases: Web of Science, Educational Resource Information Centre (ERIC), Scopus, and Linguistics & Language Behavior Abstracts (LLBA). Two categories of search terms were created. The first included the keyword “positive psychology” and a range of PP‐related terms, such as “happiness science” and “positive emotion.” The second set of terms included combining the keyword “second language” using modifiers “second language” and “foreign language” to be specific in SLA studies. Searches were performed in the title, abstract, and keyword fields, and the two sets of terms were combined using Boolean operators (OR and AND) to retrieve studies related to PP in L2 learning and teaching. Double quotation marks were used to ensure exact phrase matching, while truncation symbols were applied where appropriate to capture spelling variations. The search terms included: positive psychology (“positive psychology” OR “happiness science” OR “flourishing” OR “gratitude” OR “hope” OR “subjective well‐being” OR “flow” OR “grit” OR “resilience” OR “self‐compassion” OR “positive emotion*” OR “mindful*”), and second language (“second language” OR “L2” OR “foreign language” OR “FL” OR “ESL” OR “EFL”). For reproducibility purposes, the complete database‐specific search syntax is presented in Appendix 1.

### 2.2 Inclusion and Exclusion Criteria

The inclusion and exclusion criteria for selecting studies on PP in SLA are presented in Table 2.

**TABLE 2:** Inclusion and exclusion criteria.

	Inclusion criteria	Exclusion criteria	Rationale
(1) Focus	Studies that explicitly examine, synthesize, conceptualize, operationalize, or apply PP theories, frameworks, models, constructs, or interventions (e.g., well‐being, grit, flow, resilience, enjoyment, emotion regulation, and loving pedagogy) in SLA.	Studies not grounded in PP theories, frameworks, or constructs, or studies that mention them only in passing without clear theoretical, conceptual, or analytical relevance.	To ensure conceptual alignment with the review's focus on PP in SLA.
(2) Study type/design	Systematic reviews, meta‐analyses, theoretical papers, conceptual models, and empirical studies using quantitative, qualitative, or mixed‐methods designs to explore PP in SLA.	Opinion pieces, editorials, book reviews, workshop papers, conference abstracts, and conference papers.	To capture both higher‐level theoretical syntheses and recent empirical applications relevant to PP in SLA.
(3) Learning scope	Studies involving any aspect of SLA or L2 learning/teaching (e.g., vocabulary, speaking, writing, motivation, classroom emotions, engagement, teacher‐related variables, or instructional interventions) in which PP is a core focus.	Studies unrelated to SLA (e.g., L1 acquisition, general psychology, workplace well‐being, or teacher well‐being not clearly tied to L2 learning/teaching).	To maintain thematic relevance to L2 learning and teaching processes.
(4) Publication type	Peer‐reviewed articles published in SSCI‐indexed journals.	Articles published in non‐SSCI journals.	To ensure a clearly bounded corpus of peer‐reviewed articles published in internationally indexed journals.
(5) Publication date	Review articles, theoretical papers, and conceptual papers published between 2021 and 2025; empirical studies published in 2025.	Review articles, theoretical papers, and conceptual papers published before 2021; empirical studies published before 2025.	To adopt a two‐stage design that captures recent higher‐level theoretical syntheses while also identifying the most up‐to‐date empirical applications of PP frameworks in SLA.

The differing timeframes for review articles and empirical studies were purposively designed. Review articles published between 2021 and 2025 were used to capture higher‐level theoretical syntheses in the field, whereas empirical studies published in 2025 were selected to reflect the most recent applications of these frameworks in current research practice. This two‐stage design enabled the review to combine retrospective theoretical consolidation with a forward‐looking analysis of emerging empirical trends.

### 2.3 Data Charting and Coding Procedure

Two independent coders completed two calibration rounds and piloted five studies to finalize the codebook, including coding units, decision rules, and representative examples. After calibration, the two coders independently assessed the remaining studies, with an initial absolute agreement rate of 95%. Discrepancies were cross‐checked and resolved through discussion until consensus was reached.

A structured coding scheme (see Appendix 2) was established prior to analysis to ensure systematic and transparent data extraction. The coding scheme covered bibliographic information, contextual features, methodological characteristics, and substantive findings. Specifically, the coded items included publication year; journal title, category, ranking, and impact factor; research region; study background or motivation; research gap; research questions; theoretical framework(s) and/or model(s); study type; sample size; participant age; data collection method(s); and principal findings.

Each coding item was defined in advance using explicit operational criteria. The coding of theoretical frameworks and models followed especially strict decision rules. A theory/model was coded only if it was explicitly identified in the study and played a substantive role in framing the research, guiding the analysis, or interpreting the results. References cited merely as general background were excluded from theory coding. For review studies, theories/models used by the review authors themselves were distinguished from those summarized from the included empirical studies. For empirical studies, all explicitly stated theories/models were coded separately and subsequently classified into broader conceptual domains. Where a study drew on multiple frameworks, all relevant frameworks were retained in the coding. Any uncertain cases were resolved through discussion between coders until agreement was achieved.

### 2.4 Data Analysis

The analysis proceeded in two stages. First, recent review, theoretical, and conceptual articles on PP in SLA were screened to identify higher‐level theoretical discussions. Second, empirical studies using quantitative, qualitative, or mixed‐methods designs were examined in detail.

Descriptive content analysis with categorical coding and frequency‐based synthesis was used to map the included studies. This approach was appropriate for this study because it enabled the identification of patterns (e.g., theoretical frameworks and methods) across heterogeneous studies (Arksey and O'Malley 2005). For each of the two phases, the following were analyzed: (1) publication year, (2) publication journal, journal quality, and Journal Impact Factor (JIF), (3) geographical distribution, (4) research nature and data collection methods, (5) theoretical models and/or theoretical frameworks used, and (6) categorization of such theoretical models and/or theoretical frameworks.

After data extraction, the identified theories and models were compared in terms of their conceptual focus, explanatory mechanisms, level of analysis, and operationalization in SLA. The analysis was primarily descriptive and interpretive, consistent with the aims of a scoping review. The identified theories and models were compared in terms of their conceptual focus, explanatory mechanisms, level of analysis, and typical operationalization in SLA contexts. This process led to the development of an author‐generated hierarchical classification of theoretical frameworks used in PP–SLA research.

## 3 Results and Discussion

### 3.1 Description of Included Studies

**FIGURE 1:**
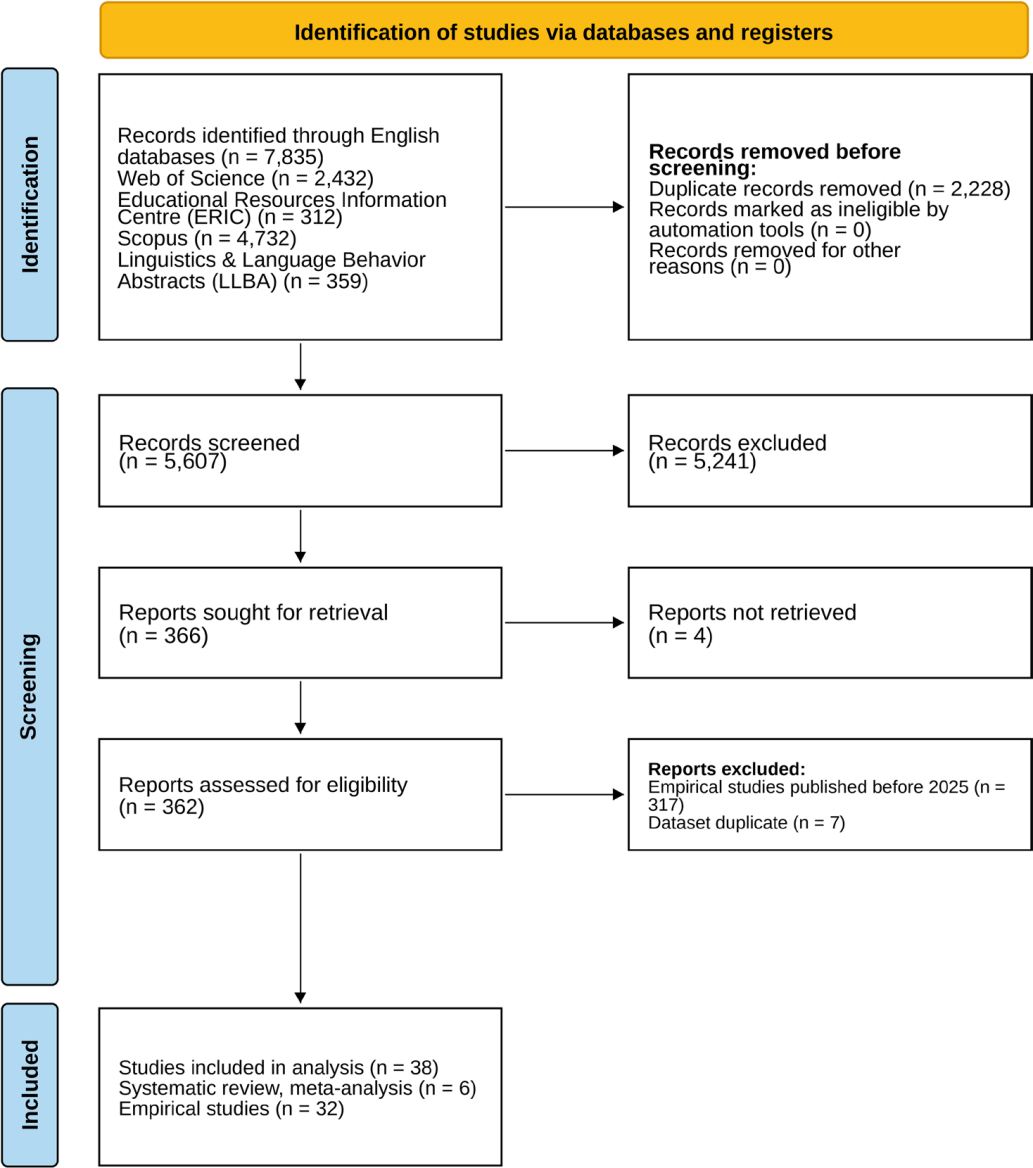
PRISMA‐ScR flow diagram.

Figure 1 shows the PRISMA‐ScR flow diagram for study identification, screening, eligibility assessment, and inclusion. The initial search yielded 7835 records. After several screening phases, 38 studies were ultimately chosen. The final corpus comprised six review articles covering the period 2021–2025 and 32 empirical studies published in 2025.

**FIGURE 2:**
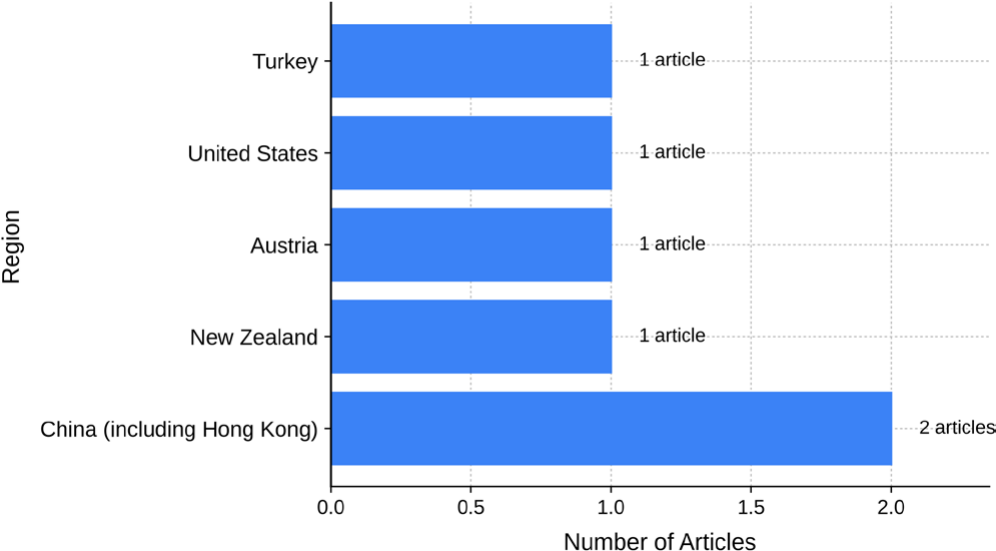
Geographic distribution of the six included review articles. The figure shows the regional distribution reported in the review articles between 2021 and 2025.

**FIGURE 3:**
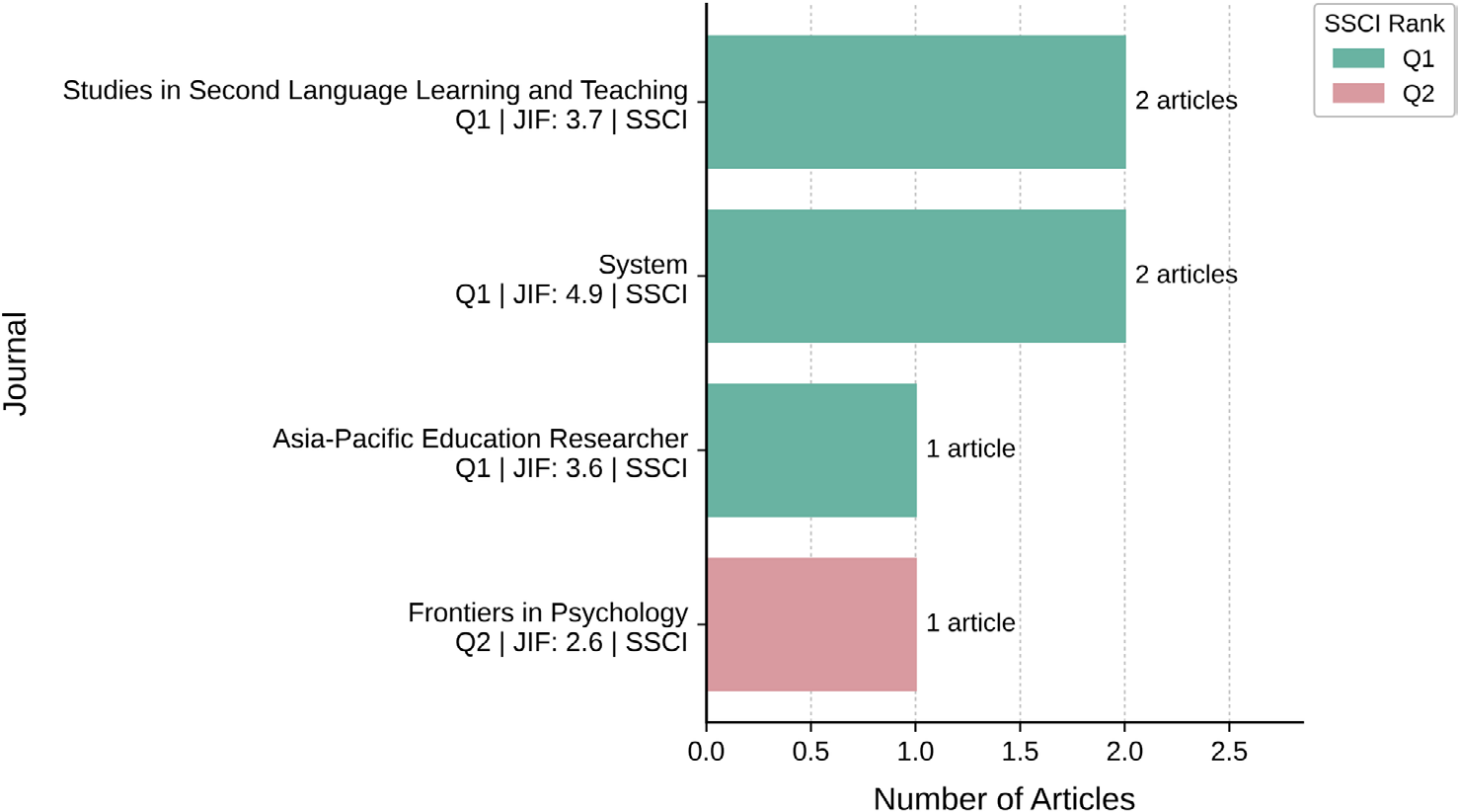
Journal information for the six included review articles. The figure presents the journals in which the review articles were published, together with their SSCI quartile rankings and Journal Impact Factors recorded at the time of data extraction.

Figure 2 indicates that review articles were written and published by Asian, Oceanian, and North American researchers, where Asia had the most reviews, with 50% of the total. Reviews were all published in high‐impact journals under the Social Sciences Citation Index (SSCI) in the Q1 and Q2 quartiles (Figure 3). In addition, according to the methodological labels and descriptions in the original published review papers, four articles were coded as meta‐analyses and two as systematic reviews.

**FIGURE 4:**
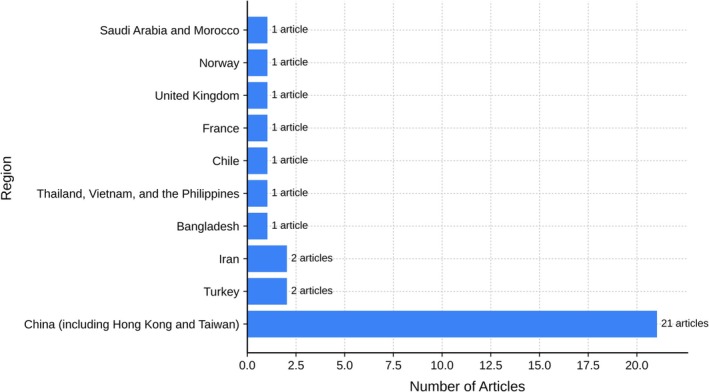
Geographic distribution of the 32 included empirical studies. The figure shows the countries or regions in which the empirical studies were conducted or where participants were located.

Figure 4 presents the geographical distribution of the 32 empirical studies. China, including Hong Kong and Taiwan, accounted for 21 studies (65.63%), followed by Iran and Turkey, each with 2 studies. Bangladesh, Chile, France, Norway, the United Kingdom, the combined Thailand–Vietnam–Philippines context, and the combined Saudi Arabia–Morocco context each accounted for one study. In terms of journal ranking, the empirical studies were concentrated in high‐impact SSCI journals: 17 studies (53.13%) were published in Q1 journals, 14 (43.75%) in Q2 journals, and only one (3.13%) in a Q3 journal. Methodologically, most empirical studies were quantitative (*n* = 23, 71.88%), particularly quantitative cross‐sectional designs (*n* = 20, 62.50%). The remaining studies were mixed‐methods studies (*n* = 8, 25.00%) or qualitative studies (*n* = 1, 3.13%). Because the journal‐quartile and methodological patterns are straightforward and primarily descriptive, they are reported narratively rather than as separate figures.

### 3.2 Major Theoretical Frameworks and Models Identified in PP–SLA Research

After a thorough review of the 38 included studies, 37 distinct theoretical models were identified (see Appendix 3). Of these, Positive Psychology (Seligman and Csikszentmihalyi 2000) was the most cited, appearing in 12 studies. It was closely followed by the Broaden‐and‐Build Theory (Fredrickson 2001), cited in 11 studies, and by both Control‐Value Theory (Pekrun 2006) and Grit Theory (Duckworth et al. 2007), each cited in 8 papers. Flow Theory (Csikszentmihalyi 1997), Self‐Determination Theory (Ryan and Deci 2000), and Social Cognitive Theory (Bandura 1986) each appeared in 6 papers. These seven theories/models were therefore selected for more detailed discussion.

#### 3.2.1 Positive Psychology

PP, with its core focus on well‐being, has had a major impact on modern theoretical frameworks regarding human flourishing (Seligman and Csikszentmihalyi 2000). As a general concept, PP is comprised of a series of models that have developed in tandem with its growth. Among them, Three Pillars (the overall architecture of PP), the PERMA model, and the EMPATHICS model representatively further our knowledge of PP. PP was treated as an overarching framework rather than a narrowly delimited theory, and it was therefore distinguished analytically from the more specific explanatory models discussed in the review.

Seligman (2002) formulated the structure of PP by Three Pillars: (1) Positive subjective experiences, for example, positive emotions and well‐being; (2) Positive individual traits, for example, emotional intelligence and resilience; (3) Positive institutions, for example, group‐level concepts like group climate and social responsibility. Subsequently, in describing SLA, MacIntyre and Mercer (2014) borrowed from three pillars to identify three applicable dimensions: (1) Positive internal learner experiences, with a focus on emotion; (2) Positive traits of learners, for example, emotional intelligence; and (3) Supportive contextual factors, for example, classroom climate.

Seligman (2011) later reconceptualized the goal of PP, shifting the focus from increasing happiness to enhancing well‐being through the PERMA model, which comprises five elements: Positive Emotion (P), Engagement (E), Relationships (R), Meaning (M), and Accomplishment (A). P is the feeling of happiness, such as satisfaction and enthusiasm. E is being fully absorbed and fulfilled in the activity itself. R is about establishing harmonious interpersonal relationships and receiving emotional support from other people. M is one's comprehension of life's purpose and dedication to purposes larger than the self. A is the perception of being competent and having confidence in achieving personal aims. It holds that each aspect, individually or collectively, accounts for overall happiness and flourishing.

Oxford (2016) further advanced the PERMA model by proposing the EMPATHICS model, an extended framework for well‐being in L2 education, incorporating affective and motivational components and emphasizing that the process of learning a language involves a complex, dynamic emotional process that inevitably involves both positive and negative emotions (Oxford 2015). EMPATHICS model includes nine interrelated dimensions: Emotional Intelligence and Emotion (E), Meaning & Motivation (M), Perseverance (P), Agency and Autonomy (A), Time (T), Habits of the mind (H), Intelligence (I), Character Strengths (C), Self‐factors (S).

Collectively, these models (i.e., Three Pillars, PERMA model, and EMPATHICS model) provide an integrated, context‐aware perspective on interpreting PP in SLA. In SLA research, this broad framework has often been operationalized through constructs such as positive L2 self, self‐efficacy, emotional intelligence, and learner well‐being. For example, Lake (2013) linked positive L2 self and self‐efficacy to intended learning effort and L2 performance, while Wang and Liu (2023) showed, through a meta‐analysis, that emotional intelligence was positively associated with L2 achievement.

#### 3.2.2 Broaden‐and‐Build Theory

Fredrickson' (2001, 218) broaden‐and‐build theory (BBT) of positive emotion suggests that good emotional states broaden “people's momentary thought‐action repertoires,” which in turn increases their scope of attention, cognitive flexibility, and behavioral responsiveness. Individuals experiencing these broadened effects tend to become more open‐minded, adaptive, and innovative, accessing more ideas and potential actions.

Specifically, the broaden aspect encompasses psychological mechanisms such as creativity, interest (including both exploration and absorption), and positive reappraisal. These mechanisms play a role in building personal resources, which Fredrickson (2001) classified into cognitive, social, psychological, and physical categories. Factors including academic performance, perceived hope, and attention all fall under cognitive resources. Social resources refer to interpersonal relationship quality, such as trust within both parent‐child relations and with peers. Individual traits like resilience, optimism, and emotion regulation are psychological resources. Physical resources include indicators of health, such as sleep hours, bodily pains, vitality, and mental health. These expanded thought‐action repertoires do not simply result in transient affectual improvement but instead underpin the accretion of long‐standing personal resources that promote long‐term growth and adaptation.

In SLA, these resources play an important role in coping with the complex cognitive and affective challenges of learning. Cognitive flexibility and positive reappraisal, for example, allow learners to reframe linguistic difficulties as growth opportunities, whereas resilience and optimism maintain motivation in encountering challenges. Social trust and interpersonal support also enhance motivation and confidence in the dynamics of classroom interaction. Therefore, BBT offers a persuasive theoretical framework for seeing the role of cultivating positive affect in improving not just learners' current affective states but also their longer‐term personal capabilities and academic achievement. To illustrate, BBT is commonly reflected in research on foreign language enjoyment (FLE), resilience, and willingness to communicate. Peng and Wang (2024) found FLE to be the best predictor of willingness to communicate in L2 use and public speaking performance. Further, positive effects make a long‐term contribution to building personal resources, which, in turn, enhance creativity, knowledge development, and social integration (Zhang and Tsung 2021).

#### 3.2.3 Control‐Value Theory

Control‐value theory (CVT) (Pekrun 2006) is an established theory in educational psychology. Within SLA, this theory was commonly adopted because of its explanatory capacity for comprehending the multidimensional nature, antecedents, and consequences, and situational specificity of achievement emotions (Li et al. 2020). It describes achievement emotions that are directly related to achievement‐related behaviors. Achievement emotions are understood as multi‐component, coordinated psychological subsystem processes that include affect, cognition, motivation, expression, as well as peripheral physiological processes (Pekrun 2006). Achievement emotions in learning environments emerge upon evaluation of events and behaviors against standards of competence (Pekrun and Perry 2014). For instance, instructional treatments and learning environments that enhance learners' perceived control and task value may elicit such positive affect states as enjoyment, pride, and interest, which in turn will fuel engagement and performance. However, students perceiving a lack of control and devaluing the task are likely to experience negative affect states such as anxiety, boredom, and hopelessness that can discourage motivation and achievement in the process of study.

**FIGURE 5:**
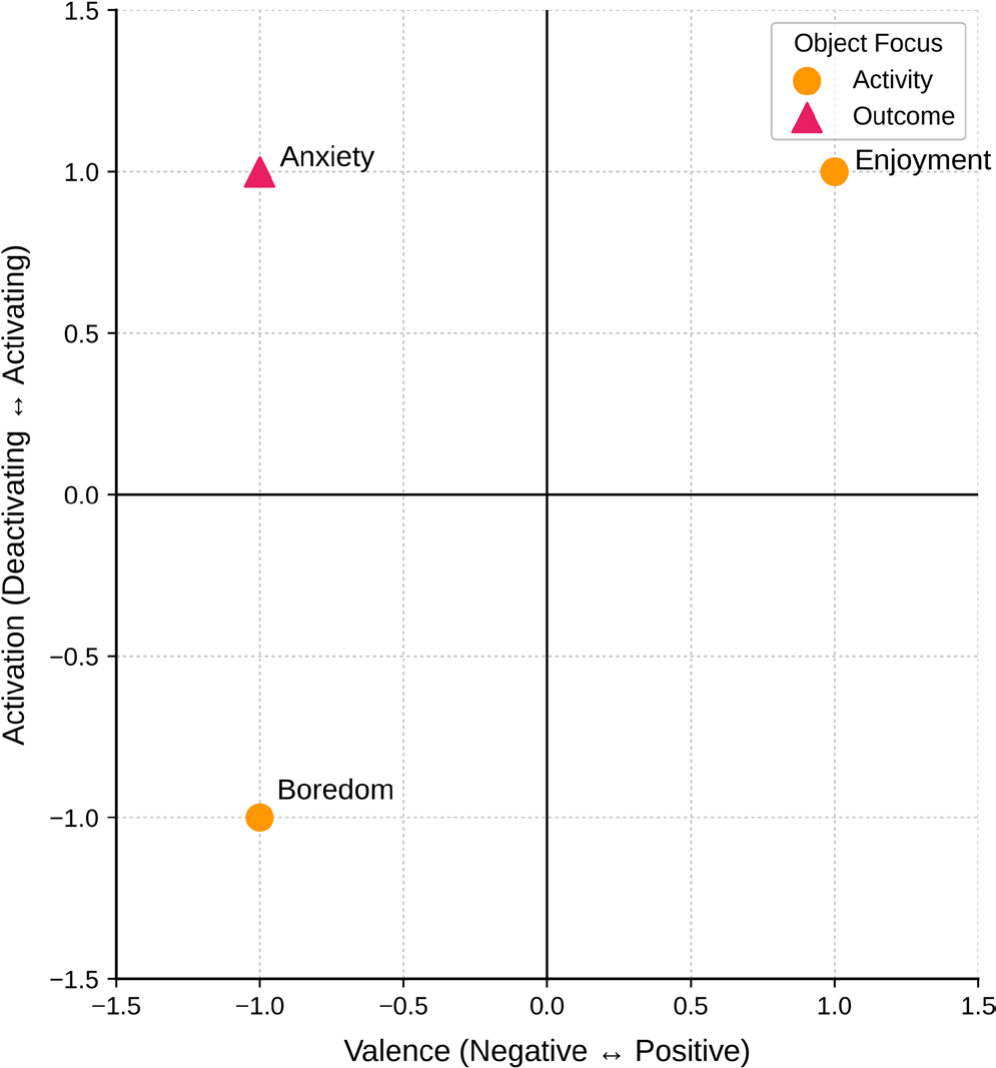
Visual categorization of emotions (informed by Pekrun 2006). Values are for illustrative purposes only.

Based on CVT, achievement emotions can be classified along three axes: valence (positive vs. negative), activation level (activating vs. deactivating), and object focus (activity vs. outcome) (Pekrun 2006). These categories explain the unique psychological functions of each emotion in performance and learning. In SLA research, CVT has most often been applied to achievement emotions such as enjoyment, anxiety, and boredom, with these constructs frequently examined using classroom‐based measures of emotional experience (Dewaele and MacIntyre 2016; Horwitz et al. 1986; Li et al. 2023). Enjoyment, for instance, is a positive, activating, activity‐centered emotion, while boredom is negative, deactivating, but also activity‐centered (Dewaele and MacIntyre 2014; Li and Wei 2025). Anxiety, by contrast, is an outcome‐centered, negative, activating emotion brought on by anticipated or recalled failure (Pekrun and Perry 2014). These differences are visually depicted in Figure 5, which plots the emotions on a coordinate plane according to valence and activation. Notably, these categories are not simply descriptive but also predictive of their roles in function in learning. According to CVT, enjoyment could enhance engagement and performance, whereas anxiety and boredom tend to hinder them (Pekrun 2006).

#### 3.2.4 Grit Theory

Grit has emerged as a distinctive and significant attribute that contributes to individuals' well‐being, such as happiness and life satisfaction in PP (Khan and Khan 2017). Grit is defined as the “perseverance and passion” for long‐term goals, originally defined as a higher‐order factor encompassing two second‐order latent factors: perseverance of effort (PE) and consistency of interest (CI) (Duckworth et al. 2007). It indicates that grittier individuals' success depends on an essential combination of long‐term dedication and persistent effort despite failures or obstacles.

Grit has gained growing attention in SLA research due to evidence supporting its positive impact on language learning outcomes (Shehzad et al. 2022). For example, research has revealed that grit is positively related to learners' willingness to communicate in an L2 environment (Lee and Lee 2020). Grit has also been reported to be associated with enhanced motivation, engagement, and English language proficiency (Zhao and Wang 2023).

In addition, grit has been developed according to different cross‐cultural views. Datu et al. (2017) introduced the Triarchic Model of Grit (TMG) and its measurement to better delineate how grit works in collectivist‐oriented cultural contexts. TMG not only adopts the original grit components of PE and CI (Duckworth et al. 2007) but adds Adaptability to Situations (AS), which means an individual's ability to modify ideas, behaviors, and strategies according to changing dynamic social, situational, and environmental environments. Collectivists prioritize interdependence and coherence at an individual's level, with the individual enveloped by the group, with priority on the “We” over the “I” (Littlewood 1999). Within societies with an emphasis on a context‐aware self (Suh 2007), AS is usually considered an indispensable resource for achievement (Datu and Zhang 2021).

Besides cross‐cultural views, grit has also been studied from a domain‐specific perspective. Researchers have increasingly argued that grit could exist differently across particular areas of achievement (Gao et al. 2025; Teimouri et al. 2020). The significant role of L2 grit in L2 acquisition has been recognized, with the empirical finding that higher L2 grit is correlated with higher L2 learning enjoyment and performance (Fathi and Hejazi 2024). This domain‐specific perspective is consistent with evidence that personality traits like grit are context‐influenced in their applicability (Cormier et al. 2019). With respect to general measurements, domain‐specific scales, such as the L2 grit scale, offer a closer approximation of learners' behaviors (Teimouri et al. 2020).

#### 3.2.5 Flow Theory

Flow refers to a state of deep engagement and concentration in which individuals feel energetic, focused, and fully absorbed in an activity (Csikszentmihalyi 1990, 1997). One characteristic of flow is control, even in situations of difficulty (Csikszentmihalyi 1990). Another characteristic of flow is extensive engagement in the task itself, which might result in a decrease in self‐consciousness (Csikszentmihalyi 1990; Chen 2007). Csikszentmihalyi (1990) characterized the sensation of losing self‐consciousness as a pleasant and soothing one.

Flow experiences can take behavioral or cognitive forms (Oliveira et al. 2022; Zhang et al. 2025). According to Zhang et al. (2025), flow manifests behaviorally in learners' active mental efforts toward the accomplishment of learning goals. Flow's typical behavioral indicators are longer study time, more academic production, and heavier concentration and attention while its cognitive indicators can be (1) rehearsal: repetition of learning content to facilitate memorization; (2) elaboration: paraphrasing or summarizing material to promote deeper understanding; (3) metacognitive: self‐regulation of one's learning process through planning and monitoring; (4) critical thinking: analyzing and evaluating new ideas critically; and (5) organizing: utilizing tables, diagrams, or charts to create and work with information more effectively (Zhang et al. 2025). These cognitive and behavioral aspects combine to promote deeper, more enduring learning engagement, typical of the flow state.

Flow has been widely recognized to be positive in learning processes. By balancing the task's difficulty and the learner's ability, flow can result in the best learning experiences (Ibrahim 2020). Researchers have indicated that flow can positively affect learners' motivation by boosting their sense of mastery and competence. In language learning, when students achieve flow, they become more engaged in the learning task, which, in turn, leads to improved retentiveness, motivation, and better performance in the target language (Liu and Song 2021).

Recent SLA research has also provided more fine‐grained evidence for the role of flow in English learning. For example, students who experience flow tend to perceive themselves as more competent and capable, which reinforces their self‐efficacy and encourages them to sustain their learning efforts (Kaya and Ercag 2023). This relationship has also been supported by recent longitudinal evidence. Jia et al. (2024), using an 8‐week weekly diary design with cross‐lagged panel modeling, found that self‐efficacy consistently predicted English as a foreign language (EFL) learners' flow experience over time, while the reverse effect was less stable. This suggests that flow should be understood not only as a desirable learning state, but also as part of a dynamic system of positive learner variables. In this sense, the engaging and reward‐driven quality of flow provides a positive feedback loop in which increased engagement strengthens learners' motivation, which in turn facilitates effective and sustained language learning. Moreover, Jia, Wang, et al. (2025) found that flow significantly and positively predicted university EFL learners' psychological capital, both directly and indirectly through academic efficacy and foreign language classroom anxiety, further indicating that flow may contribute to broader positive psychological development in language learning.

In addition, a variety of antecedents of flow have also been discovered, which explained how the optimal condition of learning can be developed. Flow's antecedents can be understood through three dimensions: behavioral, cognitive, and affective. From the behavioral perspective, the high correlation found between flow and learner engagement emphasizes the need to create language learning activities that balance the levels of challenge and skill to promote flow‐conducive environments (Özhan and Kocadere 2020). These environments have been proven to increase not only learner engagement but also the general effectiveness of learning (Hamari et al. 2016). From a cognitive view, Csikszentmihalyi (1997) indicated that flow is more probable in challenging, demanding, and goal‐oriented work, not leisure activities. Massimini and Carli (1988) also pointed out that flow arises when the perception of a balance exists both in the skills one possesses and the challenges one encounters. When the equilibrium is disturbed, most especially when the task is beyond one's capacities, learners will feel either bored or stressed. From an affective perspective in L2 learning, the high perceived usefulness of learning activities has also been linked to enhanced flow experiences (Lu et al. 2022). Learners' positive affect, including interest and enjoyment, can strengthen their intrinsic motivation and increase their engagement in learning activities, thus enhancing the flow of experience. Jia et al. (2026) further showed that self‐efficacy had a significant negative association with foreign language classroom anxiety, that flow mediated this association, and that self‐compassion moderated the relationship between self‐efficacy and flow. This finding provides timely supplementary evidence that PP constructs may work together through integrated cognitive, affective, and self‐regulatory pathways. Very recent longitudinal evidence has further shown that resilience may consistently predict ESL learners' flow experience over time, whereas the reverse effect appears less stable (Meng and Jia 2026). In sum, both theoretical and empirical research highlight a variety of flow antecedents that are critical to the development of effective language learning. These not only facilitate learner engagement but also reinforce motivation, even with problems or frustration.

In SLA research, flow has increasingly been examined as a dynamic and multidimensional construct rather than merely a momentary positive experience. For example, Jia, Meng, and Yu (2025) developed and validated an English Learning Flow Scale (ELFS) consisting of seven dimensions, namely clear goals, challenge‐skill balance, feedback, intrinsic motivation, intense concentration, control, and enjoyment, which provides a more domain‐specific measurement framework for future SLA research.

#### 3.2.6 Self‐Determination Theory

Self‐determination theory (SDT), developed by Deci and Ryan (1985) and Ryan and Deci (2000), is a comprehensive framework for understanding human motivation, particularly in social and educational contexts. SDT concentrates on the dynamic interaction between external and internal forces that shape people's behavior (Bouffard 2017). SDT focuses on three basic psychological needs (BPNs): autonomy, competence, and relatedness, which are the prerequisites of effective learning and personal development (Mulyadi et al. 2024; Ryan and Deci 2017). SDT suggests that the satisfaction of BPNs highly encourages learners to interact meaningfully with their language study programs (Ryan and Deci 2017). A review study also revealed that learners satisfied with their BPNs tend to exhibit higher levels of academic engagement and productivity (McEown and Oga‐Baldwin 2019).

SDT differentiates controlled and autonomous types of motivation, which are based on external pressures and internal values and interests, respectively. For example, learners motivated by intrinsic reasons, including personal interest or enjoyment, are likely to persevere and find satisfaction in their study (Hamm and Yeh 2024). On the contrary, extrinsically motivated learners, driven by rewards or outer goals, can accomplish certain results but not enduring persistence (Ryan and Deci 2000). In educational contexts, students' engagement and achievement quality highly depend on the quality of the motivation type in their learning situations. In SLA research, SDT is most commonly reflected in studies of autonomy, competence, relatedness, and the distinction between intrinsic and extrinsic motivation. These constructs are often used to explain learner engagement, persistence, and meaningful participation in language learning environments (Ryan and Deci 2017; McEown and Oga‐Baldwin 2019).

#### 3.2.7 Social Cognitive Theory

Bandura's (1986) social cognitive theory (SCT) is based on its core concept of reciprocal determinism, where learning is a result of bidirectional and dynamic interaction among personal factors (e.g., cognition, affect, and beliefs), behavior, and the environment. Each element influences and is influenced by all others. Reciprocal determinism in SLA foregrounds that learning is not unidirectional but rather an ongoing mechanism of feedback. Learners' beliefs, such as their self‐efficacy, can shape their behavior, which, in turn, transforms the learning environment (e.g., interaction with the teacher) and ultimately affects their motivation and emotions. Thus, SCT emphasizes the interdependence of motivation, behavior, and context and recommends an integrated perspective to understand learner development.

Additionally, studies in SLA grounded in SCT often focus on the role of self‐efficacy. Self‐efficacy is the central construct of SCT, defined as individuals' beliefs in their capacity to plan and execute the actions required to accomplish certain performance standards (Bandura 1986). According to Bandura (1997), these beliefs critically shape learners' choices, effort investment, perseverance when faced with difficulties, and eventual academic outcomes. As such, self‐efficacy serves not only as a predictor of learning success but also as a mechanism through which learners regulate their motivation and behavior.

Self‐efficacy has been found to influence achievement‐related cognitive processes and affective states in SLA. Various studies have established a positive correlation between L2 self‐efficacy and L2 achievement (e.g., Golparvar and Khafi 2021; Karbakhsh and Ahmadi Safa 2020), implying that learners with higher self‐efficacy tend to persevere more in difficult tasks and achieve greater language proficiency. Furthermore, Kitikanan and Sasimonton (2017) found a strong positive relationship between skill‐specific self‐efficacy (reading, writing, listening, and speaking) and overall L2 achievement among Thai university students. They concluded that improving self‐efficacy in a single language skill could lead to greater overall language achievement. Beyond cognitive outcomes, self‐efficacy also regulates learners' affective states. Self‐efficacy beliefs are related to perceived competence and positive feelings such as confidence (Cramer et al. 2009). Conversely, a lack of self‐efficacy is frequently associated with low perceived ability and unpleasant emotions such as worry. In SLA, SCT has been most frequently operationalized through self‐efficacy, including both general L2 self‐efficacy and skill‐specific self‐efficacy in reading, writing, listening, and speaking. Empirical studies have shown that stronger self‐efficacy beliefs are associated with higher L2 achievement and more persistent strategic engagement in language learning tasks (Golparvar and Khafi 2021; Karbakhsh and Ahmadi Safa 2020; Kitikanan and Sasimonton 2017).

### 3.3 Theory and Model Comparisons

Although all seven theories contribute to our understanding of affective and motivational processes in SLA, they differ in theoretical scope, conceptual focus, and explanatory mechanism.

First, PP, from the Three Pillars (Seligman 2002) to EMPATHICS model (Oxford 2016), provides an integrated and humanistic framework. It targets learners as thriving individuals centered upon well‐being, positive feelings, engagement in meaning‐making, and personal development. The models engage with an extensive range of affective factors, such as optimism, meaning‐making, and social connection, which were previously not considered in mainstream theories of language learning.

In contrast, CVT (Pekrun 2006) and BBT (Fredrickson 2001) take more process‐oriented and emotion‐specific perspectives. CVT clearly describes how learners' negative and positive emotions stem from appraisals of task value and control, directly situating such emotions in academic performance. BBT focuses on the functions of positive emotions to demonstrate how such emotions broaden cognitive resources and build resilience over time, thereby supporting longer‐term language development.

Grit Theory (Duckworth et al. 2007) and Flow Theory (Csikszentmihalyi 1997) are more experience‐ and performance‐focused. Grit focuses on sustained interest and long‐term persistence as central to language proficiency, especially in the context of challenges. Flow, by contrast, aims to capture learners' subjective experience of profound concentration, emphasizing optimal levels of challenge‐skill balance in inducing intrinsic motivation and enjoyment in task performance. Though both theories are concerned with sustained attention and effort, grit is concerned with long‐run behavioral habits, in contrast to moment‐to‐moment subjective experience.

SDT (Ryan and Deci 2000) and SCT (Bandura 1986) are wide‐ranging motivational theories. SDT differentiates intrinsic versus extrinsic motivation and suggests that autonomy, competence, and relatedness are necessary to maintain autonomous motivation. SCT, in contrast, emphasizes reciprocal determinism and focuses on self‐efficacy, describing how learners' beliefs about themselves interact with behavior and context to regulate patterns of behavior. Both theories combine cognitive, behavioral, and contextual variables, although SDT is more concerned with motivation quality, while SCT is more concerned with agency and regulation processes.

Although these theories all contribute to explaining affective and motivational processes in SLA, they do so from different analytical angles and therefore should be understood as complementary rather than interchangeable. One important point of integration concerns the role of emotion. For example, CVT and BBT both assign a central role to emotion, but they explain different parts of the process. CVT focuses on the antecedents of achievement emotions, arguing that learners' appraisals of control and task value give rise to emotions such as enjoyment and anxiety. BBT, by contrast, focuses on the consequences of positive emotions, showing how emotions such as enjoyment broaden learners' thought‐action repertoires and gradually build enduring personal resources such as resilience, flexibility, and social connectedness. In this sense, CVT helps explain why emotions emerge, whereas BBT helps explain what positive emotions do once they are experienced.

A similar complementary relationship can be seen between grit and SCT. Grit foregrounds long‐term perseverance and sustained interest in pursuing difficult goals, making it especially useful for explaining learners' persistence in language learning over time. SCT, however, provides a more fine‐grained account of the mechanism underlying such persistence by emphasizing self‐efficacy, agency, and reciprocal determinism between the learner, behavior, and environment. Put differently, grit describes the dispositional tendency to persist, whereas SCT explains how beliefs about capability and contextual feedback regulate that persistence in practice.

Flow Theory and SDT are also related but distinct. Flow Theory explains the subjective experience of deep absorption, enjoyment, and challenge‐skill balance during task engagement, whereas SDT focuses more broadly on the quality of motivation and the psychological conditions, namely autonomy, competence, and relatedness, that support sustained engagement. Thus, flow can be seen as an optimal experiential state during learning, while SDT helps explain the motivational conditions under which such a state is more likely to occur.

These theories are best viewed as operating at different but related levels. PP provides the broad conceptual umbrella; CVT and BBT clarify emotional antecedents and functions; Grit and Flow address persistence and engagement from trait‐like and experiential perspectives; and SDT and SCT provide broader motivational and regulatory accounts. At the same time, some tensions remain. For instance, Grit Theory tends to present perseverance as a relatively stable personal disposition, whereas SCT emphasizes the contextual and dynamic nature of self‐regulation. Likewise, BBT privileges the generative role of positive emotions, whereas CVT incorporates both positive and negative achievement emotions within the same appraisal system. In summary, their value lies not in offering competing explanations of the same phenomenon but in jointly illuminating different dimensions of how learners feel, act, persist, and develop in SLA.

The above comparative discussion is summarized in Table 3. The table presents an author‐developed synthesis of the major theories/models identified in this review, organized by higher‐order classification, theoretical focus, core concepts, and operationalization in SLA contexts. The higher‐order classifications were developed by comparing each theory/model's conceptual scope, primary explanatory mechanism, and level of analysis, while the descriptions of each theory/model were grounded in the corresponding original theoretical sources and relevant PP‐SLA literature (e.g., Seligman and Csikszentmihalyi 2000; Fredrickson 2001; Pekrun 2006; Ryan and Deci 2000; Wang et al. 2021).

**TABLE 3:** Classification and operationalization of major theoretical frameworks in PP–SLA research.

	Classification	Theory/model	Focus	Core concepts	Operationalization in SLA
1	Well‐being and flourishing framework	*Positive Psychology* (Seligman and Csikszentmihalyi 2000)	Human development and well‐being	Positive Emotions, Strengths, Growth	Learner well‐being, positive emotions, positive L2 self, self‐efficacy, emotional intelligence, resilience, engagement, and flourishing in L2 learning
		*Three Pillars* (Seligman 2002)	Three pillars of well‐being	Positive subjective experiences, Positive individual traits, Positive institutions	Positive learner experiences, positive individual traits, and supportive classroom or institutional conditions in L2 learning settings
		b *PERMA Model* (Seligman 2011)	Five pillars of well‐being	Positive Emotion, Engagement, Relationships, Meaning, Accomplishment	Positive emotion, engagement, relationships, meaning, and accomplishment as dimensions of learner well‐being and sustained motivation in SLA
		c *EMPATHICS Model* (Oxford 2016)	Positive psychology in educational contexts	Emotion, Meaning, Perseverance, Agency, etc.	Emotion, meaning, perseverance, agency, time, habits of mind, intelligence, character strengths, and self‐related factors in L2 learning
2	Emotion‐centered theories	*Broaden‐and‐Build Theory* (Fredrickson 2001)	Function of positive emotions	Broadening cognition, building personal resources	Foreign language enjoyment, interest, optimism, resilience, willingness to communicate, cognitive flexibility, and learners' capacity to reframe language‐learning challenges
3		*Control‐Value Theory* (Pekrun 2006)	Achievement emotions and regulation	Control appraisal, Value appraisal, Emotion regulation	Control appraisals, value appraisals, and achievement emotions such as enjoyment, anxiety, boredom, pride, and hopelessness in L2 classroom learning
4	Persistence and engagement theories	*Grit Theory* (Duckworth et al. 2007)	Long‐term effort and persistence	Perseverance, Passion for long‐term goals	Perseverance of effort, consistency of interest, L2 grit, and, in some cultural contexts, adaptability to situations
5		*Flow Theory* (Csikszentmihalyi 1997)	Optimal learning experience	Deep engagement, Challenge–skill balance	Challenge–skill balance, clear goals, feedback, intrinsic motivation, intense concentration, sense of control, enjoyment, and task absorption during L2 learning activities
6	Motivational regulation theories	*Self‐Determination Theory* (Ryan and Deci 2000)	Types and quality of motivation	Autonomy, Competence, Relatedness	Autonomy, competence, relatedness, intrinsic motivation, extrinsic motivation, and basic psychological need satisfaction in L2 learning environments
7		*Social Cognitive Theory* (Bandura 1986)	Social–cognitive interaction and agency	Self‐efficacy, Agency, Reciprocal determinism	General and skill‐specific L2 self‐efficacy, learner agency, strategic engagement, and reciprocal interactions among learner beliefs, behavior, and classroom environment

*Note:* This table presents an author‐developed synthesis of the theories/models identified through the coding process. Higher‐order categories are generated by comparing each theory/model's conceptual scope, primary explanatory mechanism, and level of analysis. The operationalization column summarizes how each framework has typically been translated into constructs, variables, or dimensions in SLA contexts based on the reviewed studies and relevant foundational sources.

## 4 Future Directions

In addition to these breakthroughs, several methodological and theoretical gaps still exist. First, existing frameworks are predominantly neutral in age, with few age‐specific implementations that consider developmental, cognitive, and emotional differences among children, adolescents, and adults. Developing age‐specific PP frameworks would allow us to consider learners' changing emotional and motivational states throughout their lifespan.

Second, although theories such as SDT have been helpful in formal classroom environments, their extension to non‐formal, digital, and technology‐enhanced learning contexts remains underdeveloped. Ryan and Deci (2020) suggest that understanding how newer technologies meet BPNs is essential to deepen learner engagement in more recent environments. Future studies may further examine how emerging technologies, including AI‐supported tools, can support learners' psychological needs, positive emotions, and sustained engagement.

Third, because most PP models originated in Western cultures, their applicability to other sociocultural settings requires further scrutiny. Greater attention should therefore be paid to cross‐cultural comparisons and contextual adaptation, especially in East Asian and other non‐Western educational environments where emotional norms, teacher–student relationships, and values surrounding learning may differ in important ways (Gao et al. 2025). In this regard, Wang et al. (2021) also emphasized the importance of extending PP research in L2 learning and teaching toward broader contextual, pedagogical, and cross‐cultural inquiry.

Fourth, the current study primarily focuses on learners, whereas teacher‐related dimensions in PP research deserve much greater attention. Recent evidence suggests that variables such as loving pedagogy, emotion regulation, and self‐efficacy are closely related to teachers' work engagement across different EFL contexts (Wang et al. 2025). In addition, teacher development should be more explicitly addressed, as self‐directed professional development has been shown to bring both benefits and challenges for Chinese EFL pre‐service teachers (Wang and Wang 2023). More broadly, PP research in second/foreign language learning and teaching should further extend toward teacher education, cross‐cultural inquiry, and pedagogical application (Wang et al. 2021). Future research should therefore integrate teacher perspectives more fully and explore how PP frameworks can guide teacher development and practice across diverse contexts.

Fifth, future PP–SLA research should make greater use of longitudinal and experimental designs (Khajavy et al. 2025). The current empirical evidence base remains largely dominated by cross‐sectional quantitative studies, which limits the field's ability to examine temporal ordering, developmental change, and causal mechanisms. Longitudinal panel studies, diary studies, experience‐sampling designs, classroom‐based interventions, and randomized or quasi‐experimental studies would allow researchers to test how PP constructs evolve over time and whether PP‐informed pedagogical supports produce sustained gains in both language learning outcomes and learner well‐being.

## 5 Conclusion

This scoping review mapped the use of theoretical frameworks in PP research in SLA and showed that the field is supported by a diverse but uneven theoretical base. Its central contribution lies in proposing a hierarchical classification of framework use. At the broad level, PP provides the overarching humanistic orientation. At a more specific level, BBT and CVT explain the antecedents and functions of emotions. Grit Theory and Flow Theory account for persistence and optimal engagement. SDT and SCT provide broader accounts of motivational regulation, psychological need satisfaction, self‐efficacy, and learner agency. By organizing these theories according to conceptual focus, explanatory mechanism, and typical SLA operationalization, this review moves beyond a descriptive list of theories and offers a structured map of how PP–SLA research is theoretically organized. This classification can support future researchers in selecting more appropriate frameworks, designing theory‐informed empirical studies, and clarifying how PP constructs function in different L2 learning and teaching contexts. Taken as a whole, these models complement Maslow's (1943) observation. In SLA, this emphasizes language learners' profound human tendency to actualize themselves through language, development of selves, as well as communication across cultures. These theories indicate that language learning should be understood not only as a process of linguistic development, but also as a process of personal growth and self‐actualization.

Several limitations should also be acknowledged. First, the review adopted a scoping rather than a conventional systematic review design. Therefore, it did not include a formal methodological quality appraisal of all included studies. Second, the two‐stage sampling strategy used different timeframes for review/theoretical articles and empirical studies, which was appropriate for mapping both recent theoretical syntheses and current empirical applications, but may not capture all historical developments in the field. Third, the review was limited to SSCI‐indexed journal articles and English‐language database searches, which may have excluded relevant work published in other languages, books, edited volumes, or non‐SSCI journals. Finally, the operationalization of theories was coded based on how the original authors explicitly reported and used theoretical frameworks, meaning that implicit or underdeveloped theoretical influences may not have been fully captured.

Nevertheless, this review makes several contributions to the growing field of PP in SLA. Theoretically, it goes beyond listing individual theories by showing that the field is structured around an overarching PP perspective together with a smaller group of more specific explanatory theories/models, including BBT, CVT, Grit Theory, Flow Theory, SDT, and SCT. By comparing these theories across scope, mechanism, and level of analysis, the review clarifies how they complement one another in explaining learners' affective, motivational, and developmental processes.

Practically, the scoping review provides a clearer conceptual map of PP in SLA and identifies important directions for future theory building, empirical design, and pedagogical application. These include age‐sensitive theorization, the extension of PP frameworks to digital and non‐formal learning environments, the need for stronger cross‐cultural validation, and the integration of teacher‐related perspectives into PP‐informed SLA research. Taken together, these contributions provide a clearer basis for future theory building, empirical design, and educational application in the study of PP in SLA.

## Funding

This work was supported by Universidade de Macau, MYRG‐GRG2024‐00137‐FED, MYRG‐GRG2025‐00316‐FED.

## Ethics Statement

The authors have nothing to report.

## Conflicts of Interest

The authors declare no conflicts of interest.

## Data Availability

The datasets used and/or analyzed during the current study are available from the corresponding author upon reasonable request.

## Appendix 1 Full Database‐Specific Search Syntax

**Table:**

Databases	Search syntax
Web of Science	TS = ((“positive psychology” OR “happiness science” OR flourishing OR gratitude OR hope OR “subjective well‐being” OR flow OR grit OR resilience OR “self‐compassion” OR “positive emotion*” OR mindful*) AND (“second language” OR “second language acquisition” OR SLA OR L2 OR “L2 acquisition” OR “L2 learning” OR “foreign language” OR “foreign language learning” OR FL OR ESL OR EFL))
ERIC	TI (“positive psychology” OR “happiness science” OR flourishing OR gratitude OR hope OR “subjective well‐being” OR flow OR grit OR resilience OR “self‐compassion” OR “positive emotion*” OR mindful*) AND TI (“second language” OR “second language acquisition” OR SLA OR L2 OR “L2 acquisition” OR “L2 learning” OR “foreign language” OR “foreign language learning” OR FL OR ESL OR EFL) OR AB (“positive psychology” OR “happiness science” OR flourishing OR gratitude OR hope OR “subjective well‐being” OR flow OR grit OR resilience OR “self‐compassion” OR “positive emotion*” OR mindful*) AND AB (“second language” OR “second language acquisition” OR SLA OR L2 OR “L2 acquisition” OR “L2 learning” OR “foreign language” OR “foreign language learning” OR FL OR ESL OR EFL) OR KW (“positive psychology” OR “happiness science” OR flourishing OR gratitude OR hope OR “subjective well‐being” OR flow OR grit OR resilience OR “self‐compassion” OR “positive emotion*” OR mindful*) AND KW (“second language” OR “second language acquisition” OR SLA OR L2 OR “L2 acquisition” OR “L2 learning” OR “foreign language” OR “foreign language learning” OR FL OR ESL OR EFL)
Scopus	TITLE‐ABS‐KEY ((“positive psychology” OR “happiness science” OR “flourishing” OR “gratitude” OR “hope” OR “subjective well‐being” OR “flow” OR “grit” OR “resilience” OR “self‐compassion” OR “positive emotion*” OR “mindful*”) AND (“second language” OR “Second Language Acquisition” OR “SLA” OR “L2” OR “L2 acquisition” OR “L2 learning” OR “language learning” OR “foreign language” OR “FL” OR “ESL” OR “EFL”))
LLBA	TI (“positive psychology” OR “happiness science” OR flourishing OR gratitude OR hope OR “subjective well‐being” OR flow OR grit OR resilience OR “self‐compassion” OR “positive emotion*” OR mindful*) AND TI (“second language” OR “second language acquisition” OR SLA OR L2 OR “L2 acquisition” OR “L2 learning” OR “foreign language” OR “foreign language learning” OR FL OR ESL OR EFL) OR AB (“positive psychology” OR “happiness science” OR flourishing OR gratitude OR hope OR “subjective well‐being” OR flow OR grit OR resilience OR “self‐compassion” OR “positive emotion*” OR mindful*) AND AB (“second language” OR “second language acquisition” OR SLA OR L2 OR “L2 acquisition” OR “L2 learning” OR “foreign language” OR “foreign language learning” OR FL OR ESL OR EFL) OR KW (“positive psychology” OR “happiness science” OR flourishing OR gratitude OR hope OR “subjective well‐being” OR flow OR grit OR resilience OR “self‐compassion” OR “positive emotion*” OR mindful*) AND KW (“second language” OR “second language acquisition” OR SLA OR L2 OR “L2 acquisition” OR “L2 learning” OR “foreign language” OR “foreign language learning” OR FL OR ESL OR EFL)

## Appendix 2 Coding Scheme

**Table:**

Coding category	Operational definition	Decision rules
Study type	Whether the article was a review study or an empirical study.	Code each article as either review study or empirical study based on its primary design and stated purpose.
Year of publication	The year in which the article was officially published.	Record the publication year as reported in the final published version of the article.
Journal information	Bibliographic and quality‐related information about the journal in which the study was published.	Record the journal title, journal category, journal ranking, and journal impact factor (JIF), where available. If a value was unavailable, mark it as not reported.
Region	The geographical context associated with the study.	For empirical studies, code the country or region where the research was conducted or where participants were located. For review studies, code the region if the review explicitly focused on a particular country, region, or geographical context; otherwise the first author's was coded.
Background/research motivation	The problem, context, or rationale that motivated the study.	Summarize the main background or motivation stated by the authors. Code only information explicitly presented in the introduction, background, or rationale sections.
Research gap	The specific gap, limitation, or underexplored area identified by the authors.	Code the research gap only when it was explicitly stated or clearly identifiable from the authors' problem statement. Do not infer a gap unless it is strongly supported by the text.
Research question(s)	The question(s), aim(s), or objective(s) guiding the study.	Record the stated research question(s), research aim(s), or objective(s). If no formal research questions were given, extract the closest equivalent statement from the article.
Theoretical framework(s) and/or model(s)	The theory, model, or conceptual framework explicitly used to frame the study, guide the analysis, or interpret the findings.	A theory/model was coded only when it was explicitly named and functionally used in the study. General background citations without a clear theoretical or analytical role were not coded as formal frameworks. When more than one theory/model was used, each was coded separately. For review studies, theories/models used by the review itself and those reported in the included empirical studies were coded separately. For empirical studies, each explicitly stated theory/model was coded and then assigned to a broader conceptual category. Borderline cases were resolved through coder discussion until consensus was reached.
Nature of study	The methodological nature or design of the study.	For empirical studies, code as qualitative, quantitative, or mixed methods according to the dominant or stated design. For review studies, code according to the review type when specified (e.g., systematic review, scoping review, meta‐analysis, and narrative review); otherwise use the most accurate description supported by the text.
Sample size	The number of participants, datasets, or included studies forming the basis of the analysis.	For empirical studies, record the number of participants or cases analyzed. For review studies, record the number of included studies if reported. If unavailable, mark as not reported.
Participant age	The age or age range of the participants involved in the study.	For empirical studies, record the reported participant age, age range, or educational stage if exact age was unavailable. For review studies, code this item only if the review explicitly specified participant age characteristics across included studies; otherwise mark as not applicable or not reported.
Data collection method	The primary method(s) used to generate or collect data.	For empirical studies, record the method(s) explicitly reported by the authors, such as questionnaires, interviews, observations, tests, experiments, document analysis, or mixed methods of data collection. For review studies, code the review's data source or evidence base only when relevant and explicitly described.
Findings	The principal results, conclusions, or patterns reported in the study.	Summarize the main findings relevant to the review purpose. Code only the authors' reported findings rather than the coder's own interpretation.

## Appendix 3 Identified Theoretical Frameworks/Models and Coding Frequencies

**Table:**

No.	Theoretical framework/model	Broader conceptual domain	Frequency
1	Positive Psychology	Well‐being and flourishing framework	12
2	Broaden‐and‐Build Theory	Emotion‐centered theory	11
3	Control‐Value Theory	Emotion‐centered theory	8
4	Grit Theory/L2 Grit	Persistence and engagement theory	8
5	Flow Theory	Persistence and engagement theory	6
6	Self‐Determination Theory	Motivational regulation theory	6
7	Social Cognitive Theory/Self‐efficacy Theory	Motivational regulation theory	6
8	Resilience Theory/Academic Resilience Models	Persistence and engagement theory	4
9	Psychological Capital Theory	Well‐being and flourishing framework	3
10	Student Engagement Framework	Persistence and engagement theory	3
11	Willingness to Communicate Model	Motivational and socio‐psycholinguistic framework	3
12	Technology Acceptance Model/Integrated Model of Technology Acceptance	Technology and digital learning framework	3
13	PERMA Model of Positive Psychology	Well‐being and flourishing framework	2
14	Self‐Regulated Learning Theory	Motivational regulation theory	2
15	Emotion Regulation Framework	Emotion‐centered theory	2
16	Dynamic Systems Theory/Complex Dynamic Systems Theory	Dynamic and developmental framework	2
17	Maslach Burnout Model	Teacher well‐being and occupational psychology framework	2
18	Three Pillars of Positive Psychology	Well‐being and flourishing framework	1
19	EMPATHICS Model of Positive Psychology	Well‐being and flourishing framework	1
20	Mindfulness Theory	Well‐being and self‐regulation framework	1
21	Emotional Intelligence Theory	Emotion‐centered theory	1
22	Proactive Language Learning Theory	Motivational regulation theory	1
23	Ecological Systems Theory	Socio‐contextual framework	1
24	Sociocultural Theory	Socio‐contextual framework	1
25	Humanistic Educational Theory	Well‐being and educational framework	1
26	European Digital Competence Framework	Technology and digital learning framework	1
27	Multimedia Learning Theory	Cognitive learning framework	1
28	Emotional Design Theory	Emotion‐centered and learning design framework	1
29	Classroom Environment Theory	Socio‐contextual framework	1
30	Language Mindset/Growth Mindset Theory	Motivational and cognitive framework	1
31	Gardner's Socio‐educational Model/Motivation Intensity	Motivational regulation theory	1
32	Dynamic Model of Multilingualism	Sociolinguistic and cognitive framework	1
33	Proactive Personality Theory	Personality and motivational framework	1
34	Work Engagement Model	Teacher‐related positive psychology framework	1
35	Relational Theory	Interpersonal and socio‐emotional framework	1
36	Rhetorical/Relational Goal Theory	Interpersonal communication framework	1
37	Emotional Crossover Theory	Emotion‐centered interpersonal framework	1

*Note:* Frameworks/models were counted only when they were explicitly identified in the included study and played a substantive role in framing the research, guiding the analysis, or interpreting the findings. Because some studies employed multiple frameworks, the frequency counts represent framework occurrences rather than mutually exclusive study counts. Purely methodological or statistical procedures, such as design‐based research and grounded theory procedures, were not counted as theoretical frameworks unless they were used as substantive conceptual models.
